# Nature’s Remedies: Exploring the Potential of Propolis to Alleviate Non-Motor Manifestations of Parkinson’s Disease

**DOI:** 10.3390/molecules30081672

**Published:** 2025-04-08

**Authors:** Kételin Vitória Matias, Valeria de Cassia Gonçalves, Fulvio Alexandre Scorza, Josef Finsterer, Rozana Mesquita Ciconelli, Carla Alessandra Scorza

**Affiliations:** 1 Disciplina de Neurociência, Departamento de Neurologia e Neurocirurgia, Universidade Federal de São Paulo (UNIFESP), São Paulo 04039-032, SP, Brazil; ketelin.matias@unifesp.br (K.V.M.); vaal.cassia@gmail.com (V.d.C.G.); scorza@unifesp.br (F.A.S.); 2 Departamento de Pesquisa da BP, A Beneficência Portuguesa de São Paulo, São Paulo 01323-001, SP, Brazil; rozana.ciconelli@bp.org.br; 3 Neurology and Neurophysiology Center, 1180 Vienna, Austria; fifigs1@yahoo.de

**Keywords:** Parkinson’s disease, non-motor symptoms, propolis, natural compounds, neurodegeneration

## Abstract

Parkinson’s disease (PD) is a complex neurodegenerative disorder with debilitating non-motor symptoms, including gastrointestinal dysfunction, cardiovascular abnormalities, mood and anxiety disorders, cognitive decline, sleep disturbances, respiratory dysfunction, and pain. Despite their significant impact on quality of life, these symptoms are often inadequately addressed. Propolis is a natural bee-derived product, rich in bioactive compounds with anti-inflammatory, antioxidant, immunomodulatory, and neuroprotective properties, which holds potential in PD due to its multitarget and multipathway actions, addressing various underlying mechanisms of non-motor symptom diseases. Preclinical and clinical studies suggest that propolis may influence key pathological mechanisms in PD’s non-motor symptoms. Evidence points to its potential benefits in improving cognition, mood disorders, gastrointestinal health, and alleviating cardiovascular and sleep-related issues. Although research on propolis in non-motor symptoms of PD remains scarce, findings from related conditions suggest its ability to influence mechanisms associated with these symptoms. This review underscores the underexplored therapeutic potential of propolis in non-motor symptoms of PD, drawing on existing evidence and advocating for further research to fully assess its role in addressing these symptoms and improving patient outcomes.

## 1. Introduction

Neurological disorders account for a substantial share of disability worldwide, with Parkinson’s disease (PD) standing out as the most rapidly increasing brain disorder, posing significant challenges to global public health systems [1]. Over the past 25 years, the prevalence of PD has doubled, a trend influenced by demographic shifts toward an aging population, improved life expectancy, and heightened exposure to environmental factors such as pesticides, industrial pollutants, and other chemical agents [1,2,3,4]. Genetic research has underscored the multifactorial nature of PD. While monogenic forms account for approximately 3–5% of cases, the majority are sporadic or idiopathic, arising from a complex interplay between genetic predispositions and environmental influences [5,6,7].

**Figure 1:**
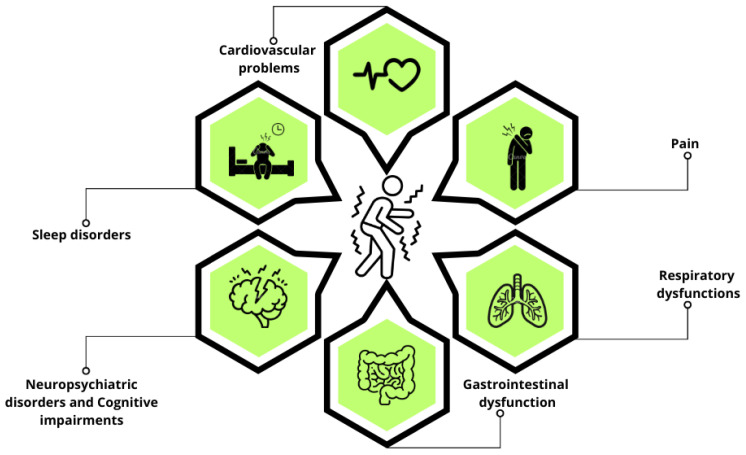
Non-motor symptoms in PD. This schematic representation highlights the primary non-motor symptoms of PD, including sleep disorders, neuropsychiatric disorders, cognitive impairments, cardiovascular problems, pain, gastrointestinal dysfunction, and respiratory dysfunctions.

PD has long been understood as a neurodegenerative condition primarily defined by motor symptoms like bradykinesia, tremors, and rigidity. Although these hallmark motor features are widely acknowledged, non-motor symptoms—such as autonomic dysfunction (Figure 1), mood and anxiety disorders, gastrointestinal issues, pain, and sleep disturbances—are equally common and can profoundly affect patients’ quality of life [6,8,9,10]. Non-motor symptoms can manifest years before the onset of motor symptoms, and in some patients, they may remain the predominant features of the disease [11,12]. These prodromal non-motor symptoms can appear up to 20 years before a clinical diagnosis, which typically focuses on motor dysfunction [10,13]. Identifying biomarkers for the prodromal stage of the disease remains a critical challenge, as this would pave the way for interventions aimed at altering the course of the disease. Due to this complexity, PD is now recognized as a heterogeneous disorder, encompassing a spectrum of subtypes with unique etiologies, clinical presentations, and progression patterns [6,14]. Understanding these nuances is essential for advancing the development of more targeted and effective therapeutic strategies.

To date, there is no cure for PD, nor any treatment that can slow or halt its progression [15]. While levodopa remains the gold standard for the symptomatic management of motor dysfunction, the degree of response can vary widely. Some patients experience significant improvements in motor symptoms, while others have a limited or fluctuating response. Current therapies primarily address motor aspects, offering little relief for non-motor issues [16,17]. Despite their profound impact on patient outcomes, non-motor features remain an underrecognized and undertreated component of the disease, leaving a significant gap in patient care. The complexity and subtle nature of non-motor symptoms, combined with the lack of targeted treatments, contribute to the difficulty in effectively managing these aspects of PD.

The key pathological feature of PD is the loss of dopaminergic neurons in the substantia nigra, accompanied by the presence of Lewy bodies, which are abnormal aggregates of the alpha-synuclein protein. However, the degree and distribution of these pathological features can vary significantly among patients [18]. Despite this variability, common underlying mechanisms, such as oxidative stress, mitochondrial dysfunction, neuroinflammation, and protein aggregation, are observed across all PD patients [6,19,20]. This multifactorial nature of PD presents a significant challenge in developing an effective therapeutic approach.

In this context, propolis emerges as a promising yet underexplored therapeutic candidate. Its pleiotropic properties enable it to modulate multiple pathological pathways simultaneously, offering a potential advantage over conventional therapies that typically target only a single mechanism. By addressing key contributors to PD pathology, propolis holds the potential to provide a multitarget approach that could more effectively address the complex and interconnected nature of the disease.

**Figure 2:**
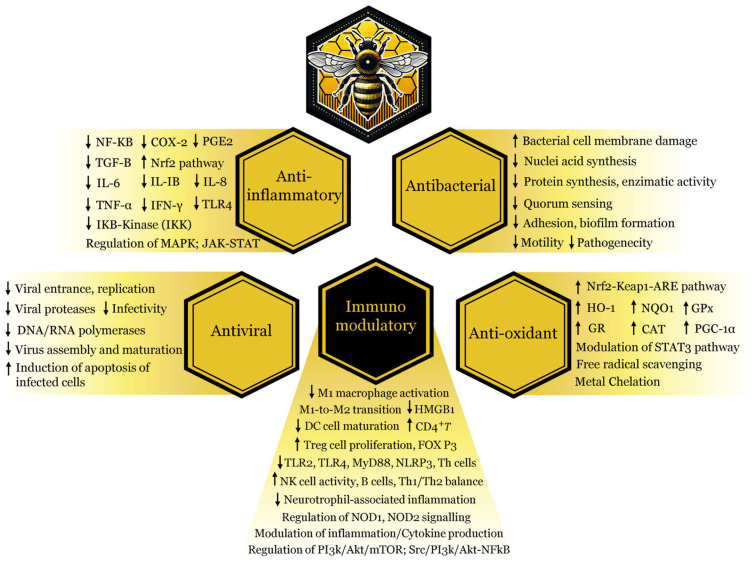
Key mechanisms underlying the beneficial effects of propolis’s bioactive compounds. The biological activity of these natural compounds is driven by anti-inflammatory, antioxidant, immunomodulatory, antibacterial, and antiviral properties. Abbreviations: NF-κB, nuclear factor kappa-light-chain-enhancer of activated B cells; COX-2, cyclooxygenase 2; PGE-2, prostaglandin E2; TGF-β, transforming growth factor beta; Nrf2, nuclear factor erythroid 2-related factor 2; IL-6, interleukin-6; IL-IB, interleukin-1beta; IL-8, interleukin-8; TNF-α, tumor necrosis factor alpha; IFN-γ, interferon-gamma; TLR4, toll-like receptor 4; IKB-kinase, inhibitory kappa B kinase beta; MAPK, mitogen-active protein kinase; JAK-STAT, janus kinase/signal transducers and activators of transcription; NRF2/Keap1-ARE, nuclear factor erythroid 2-related factor, kelch-like ECH-associated protein 1, antioxidant response element; HO-1, heme oxygenase-1; NQO1, NAD(P)H quinone dehydrogenase 1; GPx, glutathione peroxidase; GR, glutathione reductase; CAT, catalase; PGC-1α, peroxisome proliferator-activated receptor gamma coactivator 1-alpha; STAT3, signal transducer and activator of transcription 3; M1/M2, macrophages; HMGB1, high-mobility group box 1 protein; DC, dendritic cell; CD4^+^T, T-lymphocytes; Trag cell, regulatory T cells; FOXP3, forkhead box P3; TLR2/TLR4, toll-like receptor 2/4; MyD88, myeloid differentiation primary response 88; NLRP3, nucleotide-binding domain, leucine-rich-containing family, pyrin domain-containing-3; Th cells, helper T cells; NK cell, natural killer cell; B cells, B lymphocyte cells; NOD1/NOD2, nucleotide-binding oligomerization domain-containing protein 1/2; P13k, phosphatidylinositol 3-kinase; AKt, protein kinase B; mTOR, mammalian target of rapamycin; sre, sterol response element. The upward and downward arrows represent an increase or decrease in the specified components.

Propolis, a resinous substance produced by honeybees, has attracted growing interest as a potential therapeutic agent for neurological diseases due to its diverse and potent biological properties [21,22,23,24,25,26]. Rich in flavonoids, phenolic acids, terpenoids, and other bioactive compounds, propolis exhibits antioxidant, anti-inflammatory, immunomodulatory, antiviral, antibacterial, and neuroprotective effects, making it a promising natural product for scientific investigation (Figure 2) [27,28,29,30,31,32]. Research from our lab, along with findings from other studies, reveals that propolis helps preserve dopaminergic neurons and increases dopamine levels in the basal ganglia of PD animals, resulting in improved motor function [33,34,35,36,37]. Despite these promising results, there remains a notable gap in the literature regarding the impact of propolis on non-motor symptoms of PD, including gastrointestinal dysfunction, cardiovascular problems, neuropsychiatric disorders, cognitive impairments, sleep disturbances, respiratory challenges, and pain. These manifestations are often inadequately addressed by current therapies, yet they contribute significantly to disability, compromised quality of life, and reduced life expectancy.

Although most studies investigating this natural compound have not specifically focused on PD, substantial evidence supporting the beneficial properties of propolis in related conditions provides a compelling basis for exploring its potential role in managing the non-motor manifestations of PD. Given the scarcity of specific research on the direct use of propolis in addressing these symptoms, it becomes crucial to examine the state of the art of existing scientific evidence on its application in other clinical conditions and experimental models with similar signs and symptoms. By identifying and synthesizing such evidence, this literature review aims to enhance the understanding of the therapeutic potential of propolis in the non-motor symptoms of PD, highlight gaps in the current knowledge, and indicate directions for future research. Ultimately, by addressing this under-researched area, we seek to underscore the promise of propolis and its bioactive constituents, encouraging further studies that could lead to significant advancements in the treatment and management of this debilitating neurodegenerative disorder.

## 2. Results

**Figure 3:**
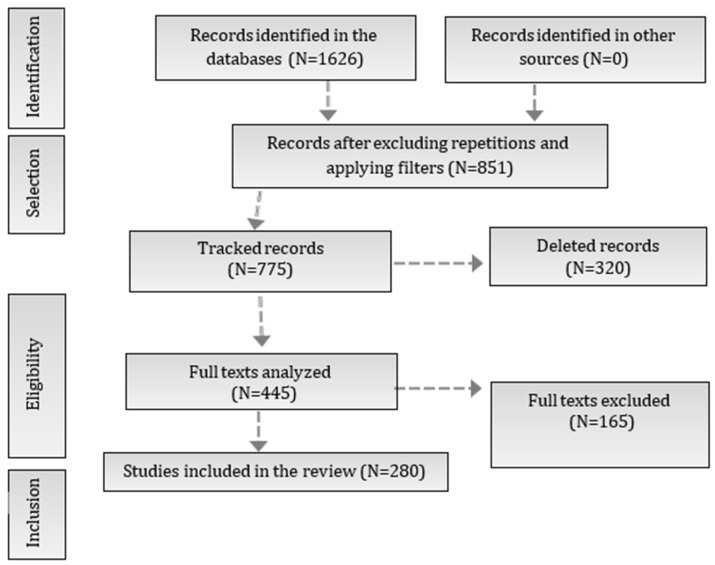
PRISMA flowchart of the study selection process. The flowchart illustrates the stages of the study selection process in the systematic review, following the PRISMA model. Initially, 1626 records were identified in the databases, of which 851 remained after removing duplicates and applying filters. From these, 775 records were tracked, leading to a full-text analysis of 445 studies. Finally, 280 studies were included in the review after excluding 165 full texts that did not meet the eligibility criteria.

The search carried out in the PubMed and Embase databases resulted in the identification of 1626 studies. After excluding duplicates and applying the inclusion and exclusion criteria, 775 records were screened. Of these, 851 records were considered eligible after excluding repetitions and applying filters. After reading all the texts, 445 articles were selected for detailed analysis. Of these, 280 studies met the inclusion criteria and were included in the final review, as illustrated in Figure 3 (PRISMA flowchart of the study selection process). The included studies were organized and synthesized according to the pathophysiological mechanisms of Parkinson’s disease (PD) and the potential therapeutic effects of propolis on non-motor symptoms. The relevant bioactive compounds and their mechanisms of action were identified and classified. In addition, three tables were drawn up: Table 1, which lists the non-motor symptoms of PD, the related diseases, the observed effects of propolis, and the bioactive compounds identified; Table 2, which presents the bioactive compounds of propolis, their mechanisms of action, the non-motor symptoms that potentially benefited, and the main associated references; Table 3, which highlights the key mechanisms underlying the benefits of propolis, relating them to the compounds involved and their respective references.

## 3. Methods

This literature review was conducted to synthesize the available evidence on the potential therapeutic effects of propolis on the non-motor symptoms of Parkinson’s disease (PD), and on different clinical conditions, with the aim of exploring its applicability in PD, particularly in its non-motor manifestations. Given the scarcity of specific studies on the use of propolis in these PD symptoms, this review sought to identify research investigating the action of propolis on pathophysiological mechanisms underlying the disease, such as neuroinflammation, oxidative stress, mitochondrial dysfunction, and the dysregulation of the neuroimmune axis, which are also involved in the manifestation of non-motor symptoms.

### 3.1. Data Sources and Search Strategy

The search was carried out in the PubMed and Embase databases, considering articles published up to 2025, in English, Portuguese, and Spanish.

To ensure the traceability and reproducibility of the search, the terms used were carefully selected and documented. The search strategy was formulated based on a combination of keywords and Boolean operators in order to identify relevant studies. The search syntax included the following terms:

#### 3.1.1. PUBMED

(Propolis OR Bee resin OR “Bee propolis”) AND (Neuroinflammation OR Oxidative stress OR “Mitochondrial dysfunction” OR “Neuroprotection” OR “Cognitive impairment” OR “Anxiety” OR “Depression” OR “Autonomic dysfunction” OR “Gastrointestinal dysfunction” OR “Pain” OR “Cardiovascular dysfunction” OR “Sleep disorder” OR “respiratory dysfunction”).

#### 3.1.2. Embase

(’propolis’/exp OR ’propolis’:ti,ab,kw OR ’bee propolis’:ti,ab,kw) AND (’nervous system inflammation’/exp OR ’neuroinflammatory diseases’:ti,ab,kw OR ’neuroinflammation’:ti,ab,kw OR ’oxidative stress’/exp OR ’oxidative stress’:ti,ab,kw OR ’mitochondrial dysfunction’:ti,ab,kw OR ’neuroprotection’:ti,ab,kw OR ’cognitive impairment’ OR ’anxiety’ OR ’depression’ OR ’autonomic dysfunction’ OR ’gastrointestinal dysfunction’ OR ’pain’ OR ’cardiovascular dysfunction’ OR ’sleep disorder’ OR ’respiratory dysfunction’).

In addition, reference articles from relevant publications were reviewed to identify additional studies not captured by the initial search.

Inclusion:

- Original articles investigating the effects of propolis in in vitro models, animals, or clinical studies.
- Studies addressing the biological mechanisms of propolis that may be related to the non-motor symptoms of PD.

Exclusion:

- Studies irrelevant to the scope of this review, such as those focused only on beekeeping aspects or diseases unrelated to PD mechanisms.

### 3.2. Study Selection Process

The selection of studies was carried out in three stages, starting with a preliminary screening based on titles and abstracts, conducted independently by two researchers. Next, the full texts of the pre-selected articles were evaluated in detail, applying the previously defined inclusion and exclusion criteria. Finally, data were extracted and analyzed from the selected publications, with special emphasis on the mechanisms of action of propolis. Agreement between the reviewers was duly assessed, and any disagreements were resolved by consensus.

### 3.3. Summary of Data

The data were qualitatively synthesized, with the evidence organized into tables highlighting the bioactive compounds in propolis and their mechanisms of action relevant to PD, as well as their effects on different clinical conditions related to the non-motor symptoms of the disease, in addition to the clinical and preclinical studies relevant to the topic. In addition, as recommended by the reviewers, a PRISMA flowchart was drawn up to clearly illustrate the article selection process.

## 4. Discussion

### 4.1. Neuropsychiatric Disorders

Anxiety and depression are common non-motor manifestations of PD and often coexist, although they can also occur independently [38,39]. Apathy is also part of the non-motor triad of the disease [40]. Studies suggest that during the prodromal phase of PD, dopaminergic depletion and serotonergic degeneration contribute to these conditions [38]. Research shows a high prevalence of mood disorders in PD patients, with depression affecting 20–50% [41,42,43,44] and anxiety disorders impacting 20–60% of patients [44,45,46], though both are often underdiagnosed and overlooked in clinical practice [10]. Anxiety and depression are associated with disruptions in the limbic-cortico-striato-thalamo circuit, which are driven by the degeneration of dopaminergic, noradrenergic, and serotonergic neurons [40]. Dysfunctions in the GABAergic and cholinergic systems also contribute to these neuropsychiatric symptoms of PD [47,48].

Inflammation is a crucial element in the pathophysiology of PD, beginning as early as the prodromal phase and contributing to the disease’s progression [31,49]. Evidence points to increased inflammatory activation as a factor in triggering depression. Research indicates that increased pro-inflammatory cytokines TNFα, IL-1β, and IL-6 levels are noted in PD [50]; their presence in peripheral blood correlates with the severity and progression of the disease. Specifically, high concentrations of IL-6 in plasma have been identified as a significant indicator of future risk for developing the disease, highlighting the crucial role of inflammation in PD pathogenesis [49,51]. Individuals with PD exhibiting more pronounced symptoms of depression, fatigue, and cognitive decline showed elevated serum concentrations of TNFα [52]. Evidence from the literature suggests that anti-inflammatory strategies may contribute to reducing the risk of neuropsychiatric disorders. For instance, a meta-analysis found that pro-inflammatory diets increase the risk of depression by 45% and anxiety by 66% compared to anti-inflammatory diets [53]. In this context, propolis emerges as a promising adjunctive therapy due to its potent anti-inflammatory properties that may help re-establish balance in the neuroimmune system.

Results from a randomized, placebo-controlled clinical trial demonstrated that adjunctive propolis therapy significantly reduces depressive symptoms in PD patients with depression [54]. A growing body of research suggests that propolis significantly alleviates depression-like and anxiety-like behaviors in PD animal models [35,55,56,57]. Propolis has been identified as a potent inhibitor of monoamine oxidase (MAO), the enzyme responsible for the breakdown of key neurotransmitters such as serotonin, dopamine, and norepinephrine in the brain [58]. These findings highlight its potential as a therapeutic option for managing PD and alleviating neuropsychiatric symptoms. Moreover, in rats subjected to a chronic unpredictable stress model of depression, propolis has been found to exhibit antidepressant-like actions by reducing oxidative stress and inflammation [59].

The medicinal properties of propolis-derived flavonoids, such as quercetin, apigenin, and chrysin, are gaining attention for their potential to alleviate neuropsychiatric disorders [60,61,62]. A study found that quercetin administration decreases brain concentrations of inflammatory factors, including IL-1β, IL-6, and NF-kB, and mitigates anxiety-like behaviors in rats with lipopolysaccharide-induced chronic neuroinflammation [63]. Preclinical studies in mice have demonstrated the antidepressant potential of apigenin, as evidenced by improved outcomes in the forced swim and tail suspension tests, possibly mediated through the modulation of the dopaminergic system [63]. Another study found that the antidepressant actions of chrysin in rodents exposed to chronic mild unpredictable stress are associated with an upregulation of brain levels of the brain-derived neurotrophic factor (BDNF) and nerve growth factor (NGF), along with its antioxidant activity [64].

The flavonoid isochlorogenic acid B in propolis helps to reduce lead-induced anxiety-like and depressive-like states in mice by reducing oxidative stress and brain inflammation while modulating the BDNF and PI3K/AKT signaling pathways [65]. A study using human neuroblastoma SH-SY5Y cells, an in vitro model for studying PD, revealed that treatment with propolis led to a significant increase in BDNF expression, mediated by the PI3K signaling pathway [66]. Research also indicates that isochlorogenic acid A reduces anxiety-like behavior in mice resulting from lead exposure by targeting neuroinflammation driven by ferroptosis through the BDNF/Nrf2/GPX4 pathways [67].

### 4.2. Cognitive Impairments

Cognition refers to the set of mental processes involved in the acquisition, processing, storage, and use of information. These processes are essential for the individual’s interaction with the environment, allowing the performance of daily tasks, problem solving, decision-making, and memory formation. Cognitive impairments in individuals with PD can manifest as difficulties in attention, memory, executive functioning, and visuospatial skills [68]. These deficits can present with significant variations in onset, characteristics, and progression rates [69]. As a major cause of disability for patients and a challenge for caregivers, cognitive impairment is observed to be up to six times more prevalent among individuals with PD than in the general population [68]. It is noteworthy that PD patients with cognitive impairment may have a significantly higher risk of developing dementia [69].

Furthermore, neuropsychiatric conditions commonly impair cognitive abilities, leading to deficits that can be both severe and complex, thereby negatively impacting patients’ quality of life. There is considerable overlap in the neurobiological mechanisms that contribute to both neuropsychiatric symptoms and cognitive impairments. Changes in neurotransmitter concentrations, such as those of dopamine, serotonin, norepinephrine, and acetylcholine, can influence mood and cognitive processes [70,71,72,73,74]. Additionally, these two aspects are affected by inflammation, oxidative stress, and neurodegenerative processes. Due to its bioactive compounds with anti-inflammatory and antioxidant properties, propolis has promising potential to counteract the biological processes that contribute to mood disorders and cognitive impairment.

A placebo-controlled, double-blind study conducted over 24 weeks in elderly Japanese participants evaluated the effects of a propolis-based dietary supplement on cognitive function [75]. The results indicated significant improvements across key cognitive domains, such as memory, attention, and information processing, highlighting propolis’s promise as an effective strategy for preserving cognitive function in the elderly [75]. In the study conducted by Zhu et al., the effects of Brazilian green propolis on cognitive decline and systemic inflammation were investigated in elderly individuals living at high altitudes (2260 m) [76]. This 24-month double-blind study involving 60 participants showed that those receiving propolis experienced significant improvements in cognitive scores (MMSE) and reductions in inflammatory cytokines, including IL-1β and IL-6, compared to the placebo group [76]. Propolis also increased levels of TGFβ1, which correlated with protection against cognitive decline compared to the placebo group, highlighting its anti-inflammatory and neuroprotective effects.

Non-pharmacological approaches, such as physical activity and nutritional interventions, combined with pharmacological treatments, have demonstrated significant benefits in alleviating both motor and non-motor symptoms of PD. These methods, being less invasive and more accessible, can effectively modulate neural activity and glial function while also reducing oxidative stress and inflammatory responses, contributing to a more balanced and comprehensive therapeutic effect [74]. In this context, propolis has emerged as a promising agent for enhancing cognitive function through multiple mechanisms, including acetylcholinesterase inhibition, the upregulation of BDNF, and a reduction in oxidative stress. The effects of propolis also lead to increased levels of brain catecholamines (norepinephrine, serotonin, and dopamine), the activation of the acetylcholine pathway, reduced neuronal loss, and the stimulation of neurogenesis [23,77].

BDNF is essential for neuron development, survival, and synaptic plasticity, playing a crucial role in the neural plasticity associated with learning and memory processes [78]. Recent studies highlight that propolis has the potential to regulate BDNF concentrations. In experiments with the SH-SY5Y cell line, a widely used model for studying PD, propolis treatment was observed to increase BDNF expression in a dose- and time-dependent manner [66]. Exhibiting adrenergic and dopaminergic traits, these cells offer a platform for studying molecular and cellular mechanisms underlying neuronal disorders, pointing to the possibility that propolis may upregulate BDNF expression and improve synaptic efficacy in human neurons [66,79].

Propolis treatment has been shown to decrease homocysteine-induced reactive oxygen species (ROS) and cell death in assays using the SH-SY5Y cell model of PD [80]. Results also revealed a reduced accumulation of cerebral protein aggregates due to hyperhomocysteinemia and improved cognitive abilities in mice on a propolis-supplemented diet [80].

The findings from Zulhendri et al. highlighted that propolis decreases neuroinflammatory and oxidative stress markers, limits leukocyte migration to inflamed areas, and reduces ROS and pro-inflammatory cytokine expression, such as IL-1β, IL-6, and TNF-α [81]. This modulation of endocrine and biochemical markers contributed to the reduction in depressive behaviors and cognitive deficits in animals. Research conducted by Zhang and colleagues investigated quercetin’s neuroprotective effects in a mouse model of memory deficits induced by paradoxical sleep deprivation (PSD). Results showed that quercetin improved memory impairment by affecting microglial activation and TLR4/MyD88/NF-κB signaling in the hippocampus [82]. As the prevalence of cognitive impairment and sleep disorders increases, these findings enhance our understanding of the underlying biological processes and open avenues for targeted interventions in conditions associated with neuroinflammation and cognitive dysfunction.

Further research into the interplay between cognitive function, mental health in PD, and the potential benefits of dietary supplements is essential to develop more effective and holistic treatment strategies and, ultimately, improve the quality of life of PD patients.

### 4.3. Sleep Disorders

The reduction in dopamine availability may be a determining factor in alterations in the circadian cycle, impacting the sleep–wake dynamics of PD patients [83]. This can result in symptoms related to sleep disturbances, a condition reported by over 60–90% of individuals diagnosed with the disease [84,85], encompassing a variety of conditions such as insomnia, circadian rhythm disorders, parasomnias, excessive daytime sleepiness, sleep behavioral disorder, rapid eye movement (REM) sleep behavior disorder, restless legs syndrome (RLS), and sleep-related breathing disorders (e.g., sleep-apnea syndrome (SAS)) [86,87,88]. Recent studies have highlighted a high prevalence of sleep-related breathing disorders in this group, including obstructive SAS, central sleep apnea, and sleep-related hypoventilation. These conditions significantly affect respiratory and circulatory functions, potentially causing a variety of systemic impacts [89].

SAS is a common condition in PD, occurring in up to 70% of patients, significantly impacting their quality of life and disease progression [89]. This frequently underrecognized condition induces intermittent hypoxia and subsequent neuroinflammation. Clinically, SAS may manifest before the motor symptoms of PD, causing recurrent upper airway obstruction, periodic breathing pauses during sleep, and sleep disruption [89,90]. A study in a mouse model of sleep apnea revealed that pinocembrin mitigates neuroinflammation by triggering BNIP3-dependent mitophagy via the JNK-ERK signaling cascade [91]. Repeated exposure to intermittent hypoxia leads to oxidative stress and inflammatory responses in brain areas associated with neurodegeneration, contributing to dopaminergic neuron loss [92]. Dopaminergic neurons in the substantia nigra are particularly sensitive to oxygen deprivation due to their higher rates of energy metabolism, basal respiration, and glycolysis, as well as increased mitochondrial populations, vulnerability to cytotoxins, and extensive axonal branching [93]. Hypoxia induces microglial overactivation, leading to the secretion of pro-inflammatory cytokines, exacerbating neurodegeneration in PD [92,94,95].

The presence of SAS increases morbidity and impairs quality of life. The pathogenesis of SAS is complex, and the therapeutic options are limited. Chronic inflammatory processes, like in PD, represent a prolonged and dysregulated immune system response to persistent stimuli, resulting in a series of negative effects on the body, including the disruption of the internal biological clock. This clock is crucial for coordinating a wide range of physiological functions throughout the day and night, encompassing sleep, wakefulness, body temperature, and hormonal regulation [96].

Chronic inflammation can negatively impact the internal biological clock in various ways. It can disrupt the production and release of neurotransmitters (e.g., serotonin and dopamine), which are essential for regulating sleep and wakefulness [97,98]. Moreover, it is associated with changes in gene expression in brain areas critical for the biological clock. These changes can prevent the synchronization of circadian rhythms with environmental light–dark cycles, resulting in irregular sleep–wake cycles and inconsistent sleep patterns [99,100].

Inflammation can also impair glial cells, which support and protect the central nervous system. The compromised function of these cells can disrupt neuronal signal transmission in the suprachiasmatic nucleus and other regions involved in the regulation of the circadian cycle [101,102,103]. Substances with anti-inflammatory, immunomodulatory, and antioxidant properties, like propolis and its constituents, may help in regulating the sleep–wake cycle.

The study conducted by Siddique and Jyoti found that transgenic flies with human alpha-synuclein aggregates in the brain, a model of PD, had a longer lifespan and higher dopamine concentrations, along with decreased oxidative stress and apoptosis, when supplemented with apigenin in their diet [104]. These findings suggest a compelling link between apigenin supplementation and enhanced dopamine levels in the Drosophila model of PD, which could have implications for sleep modulation. Dopamine concentrations are known to play a crucial role in regulating sleep–wake cycles. Additionally, the reduction in oxidative stress and apoptosis may contribute to healthier neuronal function, further supporting sleep patterns. The use of apigenin in rodents has been linked to enhanced sedation and decreased locomotor activity, both of which are key determinants of sleep latency [105,106].

Studies have highlighted apigenin as a promising candidate in modulating sleep processes and addressing sleep disturbances [107]. Apigenin, a flavonoid found in propolis, has gained interest for its anti-inflammatory, neuroprotective, and anxiolytic properties while improving neurovascular activity and concentrations of molecules such as BDNF, TrkB, and cAMP Response Element Binding (CREB), suggesting it may have a direct role in promoting sleep [107]. The potential of apigenin to improve sleep seems to be associated with its interaction with central neurotransmitter systems. Apigenin’s GABAergic activity in rats appears to work independently of GABA–benzodiazepine receptors, possibly through the GABA-A receptor mechanism [106]. This interaction is thought to facilitate an increase in inhibitory signaling, leading to sedative and calming effects that can improve sleep onset and maintenance. Evidence also suggests that apigenin amplifies sleep behaviors triggered by pentobarbital by activating chloride ion channels [108]. Moreover, the dual role of apigenin in promoting sleep and potentially attenuating age-related cognitive decline positions it as a unique molecule at the intersection of sleep and aging [107].

A study conducted by Kambe and colleagues suggests that quercetin may influence sleep architecture, partly through the activation of GABA-A receptors. The findings revealed that quercetin alters the sleep–wake cycle in rats by reducing REM sleep and increasing non-REM sleep during dark periods [109].

A study suggests that Suanzaoren Decoction, a traditional Chinese herbal remedy, may help treat sleep disorders in Parkinson’s disease. The combination of key active compounds, quercetin and kaempferol, targets important molecules such as AKT1, IL6, MAPK1, and VEGFA, potentially contributing to its therapeutic effects [110].

Apigenin, when administered in combination with a GABA-A receptor agonist, has been shown to prolong sleep duration and increase sleep rate, as well as improve sleep onset in animal models [107]. Hydroxy-cinnamic acid derivatives have been identified as agonists for GABA receptors and act synergistically with 5-hydroxytryptophan (5-HTP), both of which play a role in sleep quality [111]. These compounds can provide sedative effects, extend sleep duration, and reduce sleep latency.

Cinnamic acid (CA), a phenolic compound in propolis [112], has been explored for its antioxidant properties [113]. Its effects on circadian rhythms were assessed by monitoring wheel-running activity in constant darkness. To reduce daily fluctuations impacting the circadian clock, CA was administered continuously via intraperitoneal injection using mini pumps, addressing its short half-life. The data indicate that CA and its derivatives, like ferulic acid, may significantly influence free-running rhythms, potentially through metabolic pathways. These compounds have potential for assisting in the prevention or treatment of sleep disorders induced by environmental factors such as jet lag or shift work [114]. The inclusion of CA, along with apigenin and quercetin, in sleep disorder research could create new opportunities for developing therapeutic strategies based on natural compounds.

### 4.4. Gastrointestinal Dysfunction

Gastrointestinal dysfunction is acknowledged as a prevalent and challenging non-motor feature of PD, affecting up to 80% of patients [115,116]. Mounting evidence has highlighted the aggregation of α-syn in the enteric nervous system as a critical element in the onset and progression of PD, hinting at a potential association between gut dysfunction and the disease [117]. Braak and colleagues proposed an ’ascending anatomical theory’ after observing the dissemination pattern of α-syn aggregates, suggesting that PD advances from the GI system to the brain [118,119]. In turn, the body-first and brain-first hypotheses of PD were introduced by Borghammer and collaborators, reshaping our understanding of PD [120]. According to the body-first subtype, the disease is suggested to start in the intestine or peripheral autonomic nervous system, which could offer a more comprehensive rationale for the heterogeneity of clinical presentations observed across PD. An emerging theory underscores chronic gut pro-inflammatory processes as central, albeit silent, contributors to PD pathogenesis [121].

GI dysfunction preceding motor symptoms by as much as 20 years is well documented, with significant implications for the quality of life of individuals [122]. Within this context, constipation stands out as a common prodromal manifestation of PD. Kakino et al.’s study delineated the laxative attributes of utilizing water extract obtained from Brazilian green propolis [123]. This was demonstrated by a significant elevation in fecal mass and the restoration of stool frequency in an animal model of constipation induced by clonidine. Additionally, this intervention induced a notable augmentation in the contractile force of the isolated ileum in rodents. This enhancement was blocked by atropine, an antagonist of acetylcholine receptors, indicating that this effect is, to some extent, mediated through the activation of acetylcholine receptors [123].

Irritable bowel syndrome (IBS) without diarrhea is also recognized as a potential risk factor of PD [124,125,126]. Recent research indicated that propolis might offer utility as a supplement for individuals with IBS with constipation [127]. In this study, patients treated with propolis for a period of six weeks experienced a marked reduction in both the severity and frequency of abdominal pain in comparison to those on placebo. The effects of propolis have been attributed to its ability to aid in supporting the immune system of the GI tract and fostering beneficial gut bacteria [128]. Moreover, propolis exhibits potent antioxidant properties and can also assist in decreasing inflammation and improving nutrient absorption. Administering pinocembrin, a bioactive component found in propolis, demonstrated effectiveness against colitis by inhibiting the aberrant activation of toll-like receptor 4 (TLR4)/nuclear factor-kappa B (NF-κB) signaling in mice. Furthermore, this intervention reversed disruptions in gut microbiota and enhanced the integrity of the intestinal barrier in mice with ulcerative colitis [129].

Increasing evidence suggests that disruptions in the gut microbiome significantly contribute to the initiation and progression of PD pathology [130]. Disturbances in the symbiotic relationship between hosts and microbes result in alterations in the composition of bacteria that produce short-chain fatty acids (SCFAs) and a rise in potentially harmful intestinal pathogens [131]. As a consequence, intestinal permeability becomes compromised, facilitating the passage of microbiome-derived metabolites into the bloodstream, inducing inflammation, oxidative stress, and systemic immune reactions [132,133]. Preclinical studies have pointed out that the use of propolis can contribute to the restoration of damaged intestinal mucosa, positively impact the flora community, and boost the production of metabolites such as SCFAs [134]. SCFAs are hypothesized to have a significant role in regulating neuroimmunoendocrine functions [135]. For instance, manipulating the microbiota is posited as a potential strategy for managing PD. The expanding body of evidence proposes that propolis has the potential to enhance gut health and warrants consideration for studies investigating its utility as a prebiotic agent [136]. Propolis supplementation extends beyond the increase in SCFA content, showcasing the potential to enhance richness and diversity in gut flora, along with a reduction in both endotoxemia and inflammatory biomarkers [137]. An increasing body of research suggests that dietary propolis has favorable impacts on addressing diverse diseases by virtue of its ability to influence gut microbiota regulation [134,136,137,138,139,140].

The research undertaken by Palacios et al. yielded significant insights into the involvement of the gut microbiome in PD, particularly in its prodromal stage, involving participants with newly diagnosed PD, prodromal PD manifestations, constipation controls, and healthy individuals [141]. The study revealed a depletion in various gut butyrate-producing bacteria, known for their anti-inflammatory properties, including Anaerostipes, in both PD patients and those displaying prodromal PD features. These findings suggest that alterations in the microbiome could potentially serve as new indicators for the initial stages of PD [141]. Other studies have found evidence of a link between Anaerostipes and the risk of developing PD [142]. There is evidence indicating that fermenting propolis with the gut microbiota of obese children resulted in the enhancement of particular genera of strict anaerobes, including Anaerostipes [136]. Another investigation revealed that quercetin, a predominant flavonoid compound in propolis, reduces gut inflammation and enhances intestinal function by bolstering the proportion of SCFA-producing bacteria in lipopolysaccharide-exposed laying hens [143].

According to the latest findings, polyphenols, including those contained in propolis, are considered prebiotics as they are selectively metabolized by the host’s intestinal microorganisms and contribute to health benefits [144,145]. Propolis polyphenols may assist in fostering a favorable intestinal microbiota environment by restraining the growth of harmful bacteria and hindering the adherence of intestinal pathogens to human intestinal cells [144]. Wang and colleagues demonstrated that administering polyphenol-rich propolis resulted in positive impacts on human intestinal epithelial Caco-2 cells, a commonly utilized in vitro model for assessing intestinal barrier function [146]. The treatment resulted in a significant improvement in tight junction integrity, as evidenced by the rise in transepithelial electrical resistance and the concurrent reduction in flux rates. These regulatory effects on intestinal barrier function were partly facilitated by AMP-activated protein kinase (AMPK) and Extracellular Signal-Regulated Kinases (ERK1/2) activation and were negatively regulated by p38 MAPK signaling. AMPK acts as a key regulator of energy metabolism, and its activation strengthens epithelial barrier function and facilitates tight junction assembly [147]. ERK1/2, a member of the mitogen-activated protein kinase (MAPK) family, participates in signal transduction pathways. In turn, the p38 MAPK pathway is recognized for its involvement in relaying stress signals from the external environment [148].

In addition, Wang et al. documented that rodents fed a diet enriched with polyphenol-rich propolis showed an increased expression of the cytoplasmic scaffolding protein zonula occludens (ZO-1), a protein associated with tight junctions, in the colonic epithelium [146]. These outcomes suggest promising avenues for leveraging polyphenol-rich propolis to improve human gut health. Other investigations have also shown that polyphenolic compounds such as quercetin and kaempferol present in propolis possess the capability to enhance gut barrier function by affecting the cytoskeletal association and expression of tight junction integral membrane proteins in an intestinal epithelial barrier model [149].

The gastric pathogen *Helicobacter pylori* is implicated in chronic gastritis, ulcers, and gastric cancer, while releasing a pro-inflammatory enzyme known as urease. It has also been suggested as a potential initiator of neurodegenerative diseases [150,151,152]. Moreover, epidemiological research has described that patients with PD are at a higher risk of *Helicobacter pylori* infection [152]. Additionally, data reveal that these patients are typically older and exhibit more severe motor dysfunction, hinting at a possible association between *Helicobacter pylori* infection and the progression of PD [152]. Baltas et al. conducted research highlighting the protective properties of ethanolic propolis extracts against Gram-negative *Helicobacter pylori*. Their study revealed a notable suppression in both the growth and urease production of *Helicobacter pylori* upon propolis treatment [153]. Another study’s findings illustrated that Korean propolis possesses the capability to hinder *Helicobacter pylori* proliferation while diminishing the occurrence of its virulence elements, encompassing the cytotoxin-associated gene A responsible for encoding the urease A subunit, the surface antigen gene, and neutrophil-activating protein A [154]. Korean propolis treatment also resulted in a significant reduction in the release of pro-inflammatory cytokines and nitric oxide (NO) levels. More precisely, the findings revealed that the treatment inhibited the phosphorylation of IκBα and NF-κB p65 subunits, leading to the downregulation of their downstream target genes [154]. A mounting body of research underscores the potential of propolis supplementation as a natural means to counter *Helicobacter pylori* infection [153,154,155,156,157,158].

Investigations have pointed to a high prevalence of small intestinal bacterial overgrowth (SIBO) within the PD population [159]. Reports indicate that approximately half of PD patients exhibit SIBO, whereas its occurrence in individuals without PD stands at 8%. It raises concerns that SIBO may interfere with the absorption of medications in PD individuals [159]. Moreover, SIBO is a mechanism implicated in promoting bacterial translocation. In their research, Sabuncuoglu et al. provided evidence suggesting that propolis administration lowers bacterial translocation. This is achieved by diminishing bacterial overgrowth, improving mucosal barrier function, while reinforcing immune responses in an experimental model that affects multiple layers of gut barrier defense [160].

Despite the well-documented effects of propolis as a potent functional food capable of regulating intestinal microbiota, inflammation, metabolism, and immune response in different scenarios, its direct impact on GI disruptions in PD remains largely unexplored in scientific research. Nonetheless, given the potential implications of GI-related disturbances in PD, it is an area of interest for future research.

### 4.5. Cardiovascular Problems

Cardiac dysfunctions have been detected in approximately 80% of PD patients and can cause considerable disability and negatively impact quality of life, contributing to reduced life expectancy [56,161,162,163]. Autonomic nervous system dysfunction often occurs in PD, with damage to both the sympathetic and parasympathetic systems contributing to irregular blood pressure regulation and decreased heart rate variability (HRV), increasing the risk of cardiovascular complications [164]. Cardiac dysautonomia in PD includes symptoms such as orthostatic and postprandial hypotension, and both supine and postural hypertension [165,166]. PD patients are also more prone to ischemic heart disease, heart failure, and arrhythmias [166]. Moreover, non-dipping is highly prevalent among PD individuals, with a prevalence of up to 90% [167]. In healthy individuals, blood pressure typically decreases at night, a phenomenon known as dipping, whereas a failure to experience this nocturnal drop is referred to as non-dipping.

Additionally, sudden unexpected death in Parkinson’s disease (SUDPAR) is a critical aspect that cannot be overlooked, as it highlights the complex interplay of neurodegenerative progression, autonomic dysfunction, and potential cardiac complications associated with the disease [161,168,169,170,171]. SUDPAR is defined as an unexpected death in PD patients where no definitive cause can be determined through autopsy.

PD patients have a higher prevalence of type 2 diabetes, which increases cardiovascular disease risk [172].

Conversely, research indicates that there is an increased risk of PD in individuals with type 2 diabetes, and the potential benefit of antidiabetic medication in influencing the course of PD has been suggested [173,174,175,176,177]. While research has revealed a complex link between PD, cardiovascular diseases, and type 2 diabetes, the underlying mechanisms remain unclear; however, these may involve common factors such as disrupted lipid and energy metabolism, inflammation, and oxidative stress [178]. There is significant evidence that propolis and its bioactive constituents can counteract the primary mechanisms involved in the pathophysiology of these diseases [25,32,35,55,179,180,181].

The bioactive compounds of propolis target inflammatory pathways by downregulating pro-inflammatory cytokines via the inhibition of the NF-κB pathway and mitigating oxidative stress through the activation of the Nrf2 pathway, enhancing the activity of endogenous antioxidant enzymes [144,182,183]. The compounds in propolis also modulate critical metabolic pathways involved in glucose, insulin, and lipid metabolism [185,186,187,188]. They activate AMPK, enhancing glucose uptake and improving insulin sensitivity, and influence peroxisome proliferator-activated receptors (PPARs), promoting lipid oxidation and reducing lipid accumulation [189,190,191,192,193,194]. Propolis compounds can improve endothelial function, reduce atherosclerotic plaque formation, and attenuate myocardial oxidative damage [59,180,195,196,197,198,199,200,201,202]. Propolis and its constituents influence key signaling molecules like NO and endothelial NO synthase (eNOS), promoting vasodilation and enhancing vascular health [203,204,205,206]. Therefore, the comprehensive actions of propolis and its compounds underscore its therapeutic potential in alleviating cardiovascular complications in PD by bolstering mitochondrial function, balancing energy and lipid metabolism, reducing inflammation, and counteracting oxidative stress.

Ji and collaborators demonstrated a significant improvement in carotid obstruction, the amelioration of vascular lesions, a reduction in serum lipid profiles, and improved antioxidant activities in hypercholesterolemic rabbits following propolis administration. This effect is attributed to the downregulation or inhibition of pro-inflammatory cytokine expression, leading to the regulation of blood lipid levels and the mitigation of oxidative stress and inflammation [207]. El-Hakam and collaborators used a combination of propolis, royal jelly, and bee venom to treat hypertensive rats and noted a significant reduction in blood pressure. The flavonoids and enzymes inherent in propolis exhibit the capability to eliminate free radicals and inhibit the formation of lipid peroxides [208].

Another study found that caffeic acid phenethyl ester (CAPE), when administered to aged rats, increased the expression of antioxidant enzymes and significantly mitigated age-related ultrastructural changes in the heart and aorta [209]. Moreover, ultrastructural images of the heart and aorta showed that aged rats treated with CAPE displayed characteristics nearly identical to those observed in young rats [209]. Research findings reveal that galangin can effectively lower arterial pressure, blood glucose, insulin, and cholesterol levels in rats with metabolic syndrome [205]. Additionally, the galangin-treated group showed an increased aortic eNOS protein, elevated levels of circulating NO, and improvements in aortic endothelial dysfunction and hypertrophy. Galangin also played a role in reducing inflammation and suppressing the angiotensin II/g II/AT1R/TGF-β signaling pathway in the metabolic syndrome group [205]. Studies suggest that CAPE may serve as a promising cardioprotective agent with anti-inflammatory, antioxidant, and antiapoptotic effects, and antiarrhythmic activity [206,207,208,209,210,248,249,250,251]. However, there are significant gaps in the literature regarding the effects of propolis and its compounds on cardiovascular dysfunction in PD.

A study conducted by our research group, utilizing positron emission tomography imaging, demonstrated that rats treated with propolis exhibited increased glucose metabolism in the left ventricle compared to controls [252]. Enhanced glucose metabolism in cardiac tissue often suggests improved energy availability, which is crucial for maintaining heart function. Additionally, an untargeted metabolomics approach was employed to investigate the impact of propolis on cardiac metabolism in rats with PD, revealing its role in pathways related to oxidative stress, energy metabolism, protein and fatty acid biosynthesis, and the urea cycle [252]. Another investigation by our group showed that propolis treatment offered cardioprotective effects by countering the reductions in heart rate and HRV observed in rats with PD [35]. The examination of HRV is a non-invasive and sensitive indicator of autonomic changes. HRV reflects the capacity of the autonomic system to adapt to both external and internal stimuli, with lower HRV suggesting impaired autonomic function. Research has demonstrated that PD patients have cardiovascular changes, including decreased heart rate and HRV measurements [253,254]. Moreover, a prospective study suggested a link between decreased HRV in the non-PD population and a higher risk of developing the disease [255]. Therefore, propolis’s capacity to increase HRV suggests its potential to improve autonomic system adaptability, positioning it as a promising therapeutic agent for addressing autonomic dysfunction in PD.

### 4.6. Pain

Chronic pain is one of the most debilitating non-motor symptoms of PD. It is often underrecognized and poorly managed, affecting up to 85% of patients [256,257,258,259]. Despite its high prevalence, the mechanisms underlying pain in PD are difficult to diagnose and manage effectively. Pain in PD results from a complex interplay of central and peripheral factors, with primary causes including dopamine circuit dysfunction, altered pain pathways, increased musculoskeletal issues, disrupted inflammatory signals, and elevated oxidative stress [211,259].

Traditionally, pain in PD has been categorized based on its location and the temporal relationship to motor symptoms. However, recent advances in the understanding of pain mechanisms have led to the development of a new classification framework that emphasizes the underlying pathophysiological processes [212]. This new classification categorizes pain into three distinct types: nociceptive, neuropathic, and nociplastic, which applies when pain is neither nociceptive nor neuropathic [212,213,214,215]. This approach is in alignment with the International Classification of Diseases-11 (ICD-11), which recognizes the potential for chronic secondary musculoskeletal or nociceptive pain due to central nervous system disease. By incorporating mechanistic descriptors into the classification of pain, this framework offers a more nuanced understanding of the different pain types that may occur in PD, thus facilitating more targeted and effective treatment strategies.

Common pain types experienced by people with PD are dystonic, musculoskeletal, neuropathic, and central pain [216,217]. Given the complexity of pain and the global challenge of managing it effectively due to the adverse effects of conventional therapies like Nonsteroidal Anti-Inflammatory Drugs (NSAIDs), opioids, and opiates, there is growing interest in natural substances like propolis and its bioactive constituents, which offer analgesic, anti-inflammatory, and antioxidant properties with minimal or no side effects [218].

Propolis has been extensively studied for its analgesic and antinociceptive effects, with a substantial body of literature supporting its efficacy in alleviating pain [219,220,221]. Research consistently indicates that propolis supplementation may provide therapeutic benefits for rheumatoid arthritis patients by dampening inflammatory cascades via NF-kB pathway inhibition, decreasing ROS, malondialdehyde, and pro-inflammatory cytokines, while also enhancing antioxidants and relieving pain [222]. According to a systematic review and meta-analysis of controlled clinical trials, propolis supplementation is safe and can improve levels of glutathione (GSH), glutathione peroxidase (GPX), and total antioxidant capacity (TAC), pointing to its usefulness as an adjunct therapy in conditions associated with oxidative stress [223].

For instance, research investigating the effects of Brazilian green propolis in different mouse models of pain and inflammation revealed significant analgesic and anti-inflammatory actions, with the latter linked to the inhibition of the iNOS gene [224]. The ability of propolis to inhibit cyclooxygenase-2 (COX-2) is also crucial in its modulation of inflammatory processes, with the potential to alleviate pain and prevent the progression of inflammation-related tissue damage [225,226,227,228,229]. COX-2 is an enzyme that catalyzes the conversion of arachidonic acid into prostaglandins, which are lipid compounds that play a key role in promoting inflammation, fever, and pain. In many pathological conditions, including neurodegenerative diseases, chronic inflammation, and pain, COX-2 is upregulated, leading to the excessive production of pro-inflammatory prostaglandins.

Studies have also reported the anti-hyperalgesic effects of the ethanolic extract of propolis in chemical nociception models in rats and mice [230]. The extract of propolis has been shown to reduce neurogenic and inflammatory pain induced by the intraplantar administration of formalin, decrease paw edema, significantly inhibit capsaicin-induced pain, and reverse bradykinin-induced hyperalgesia in rodents [230]. The study by Azevedo et al. suggested that propolis flavonoids rutin and quercetin can counteract the peripheral neuropathy induced by oxaliplatin, a colorectal cancer chemotherapy agent whose use is often limited by its painful neuropathic effects. Their research found that these propolis constituents prevented neuropathy in rodents and protected dorsal horn neurons from oxidative stress, lipid peroxidation, and protein nitrosylation [231].

Evidence also indicates that CAPE, a phenolic ester compound of propolis, mitigates neuropathic pain, with its effects mainly attributed to the suppression of p38 MAPK phosphorylation, the inhibition of NF-κB translocation, and the downregulation of pro-inflammatory cytokines [232]. Research has found that CAPE relieves neuropathic pain in paclitaxel-treated rats through the inhibition of β-catenin and inflammation, along with an increase in the levels of the endogenous antioxidant Nrf2 [233].

In individuals with PD, musculoskeletal pain is linked to rigidity, reduced movement, and arthritis. CAPE reduces osteoarthritis progression through the activation of the NRF2/HO-1 pathway and suppression of the NF-κB pathway, two key regulators of oxidative stress and inflammation [234]. In response to oxidative stress or other cellular stressors, NRF2 is released and translocates to the nucleus, where it binds to antioxidant response elements (AREs) in the promoters of its target genes, such as HO-1 [235,236]. Evidence suggests that HO-1 treatment amplifies the local antinociceptive effects of opioid receptor agonists during chronic inflammatory pain in mice, involving the activation of the carbon monoxide (CO)/NO-cGMP-PKG-KATP channel’s signaling pathway [237,238,239].

Chrysin is another compound found in propolis that shows robust analgesic and antinociceptive effects. It exhibits antinociceptive actions in models of formalin-induced pain and diabetic neuropathy, with its action likely mediated by spinal opioid receptors and the CREB protein [240]. In rats with knee osteoarthritis, chrysin can alleviate neuropathic pain and reduce peripheral sensitization by inhibiting both the HMGB1-mediated activation of the RAGE/PI3K/AKT pathway and the NLRP3 inflammasome, leading to a reduction in synovitis [241]. Chrysin was found to significantly reduce heat-induced hyperalgesia and mechanical allodynia in a mouse model of experimental mononeuropathy through the involvement of serotonin 5-HT1A receptors [242]. Moreover, GABA-A receptors play a crucial role in mediating the hyperalgesic effects of chrysin observed in the tail-immersion test in mice [243].

A comprehensive examination of the analgesic and anti-inflammatory effects of propolis flavonoids such as quercetin, apigenin, luteolin, kaempferol, myricetin, hesperetin, and naringenin is documented in excellent reviews [244,245,246]. Although research on the effects of propolis and its compounds on pain in PD is still limited, current evidence highlights significant potential and growing interest in this promising field.

### 4.7. Respiratory Dysfunctions

Respiratory dysfunctions are major determinants of morbidity and mortality in PD [260]. They include a range of symptoms: respiratory dysrhythmias, tachypnea, dyspnea, sleep-related breathing disorders, and others, resulting in a complex clinical presentation that drastically reduces patient’s quality of life [261,262]. Aspiration pneumonia is cited as the leading cause of death in PD patients, primarily due to oropharyngeal dysphagia and impaired cough reflex [263].

PD-related respiratory changes are considered peripheral when associated with upper airway or breathing muscle dysfunction and central when driven by alterations in the brainstem’s respiratory control centers [260,264]. For instance, studies have shown that the neurodegeneration of medullary respiratory neurons results in respiratory impairment in PD animal models, with oxidative stress identified as a key underlying mechanism [265,266]. Given the antioxidant and anti-inflammatory properties of propolis, it could theoretically help reduce oxidative damage and protect neuronal cells within central respiratory nuclei. However, it is important to note that, despite the promising neuroprotective effects already observed in dopaminergic systems, there is currently a lack of studies directly investigating the role of propolis in the respiratory centers of the brainstem of PD patients. This points to a critical gap in the literature and could serve as a key focus for future research.

Studies showed that genistein, a phytoestrogen found in propolis, protects the genioglossus, a pharyngeal muscle essential for maintaining an open upper airway, from hypoxia-induced oxidative stress and apoptosis by engaging the PI3K-Akt and ERK1/2 MAPK signaling pathways [267,268,269]. Hypoxia is suggested as a key inducer of neuroinflammation by stimulating microglia to release pro-inflammatory cytokines [270]. Evidence points to the neuroprotective properties of Brazilian green propolis against hypoxia-driven neuroinflammation through the inhibition of microglial NF-κB activation [271]. The administration of quercetin prior to hypoxia exposure in rats reinstated the normal levels of Nrf2, HO-1, and pulmonary surfactant proteins, effectively countering the increase in inflammation, oxidative stress, and plasma protein leakage [247].

Numerous studies have highlighted the benefits of propolis and its biologically active compounds in treating respiratory tract infections [184,272,273,274]. Current research efforts have examined the anti-SARS-CoV-2 activity of propolis and its constituents [179,275,276,277,278,279]. For example, a clinical trial involving 124 patients hospitalized with COVID-19 found that those who consumed Brazilian green propolis had a shorter hospital stay [280]. Additionally, propolis may offer potential benefits by modulating the expression of transmembrane serine protease 2 (TMPRSS2), which plays a role in SARS-CoV-2 infection, and preventing the virus from binding to angiotensin-converting enzyme 2 (ACE-2) receptors, thus reducing its ability to enter cells. Propolis also shows promise in reducing inflammation through PAK1 inhibition [280].

## 5. Conclusions and Future Perspectives

While propolis and its constituents appear promising for addressing the non-motor symptoms of PD, this field of research is still underexplored. Comprehensive research is urgently needed to reveal their mechanisms of action, safety, and efficacy, which may offer new therapeutic insights and options for PD patients.

The study of natural products like propolis faces significant challenges that must be addressed to fully realize their therapeutic potential. One major limitation is the variability in propolis composition, which can result in inconsistent findings across different studies. Achieving standardization is essential to ensure consistency and reproducibility. This involves defining and controlling parameters such as the specific bioactive compounds, concentration, and extraction methods of propolis.

Another critical issue is the limited understanding of the pharmacokinetics and pharmacodynamics of propolis and its constituents. The complex mixture of bioactive compounds in propolis interacts with multiple biological targets, complicating the identification of specific mechanisms of action. Additionally, the poorly understood interactions with other nutritional supplements and conventional medications add layers of complexity to the research.

To advance the field, it is crucial to expand research involving human participants, as most studies on propolis have relied predominantly on animal models and in vitro experiments. Furthermore, the older adult population is often excluded from research, which limits the applicability of findings to this age group. Given that older adults may have different physiological responses and comorbidities, future studies should prioritize aged animals and elderly human participants to improve relevance and applicability.

Addressing the research gaps is not only important for advancing scientific knowledge but also crucial for developing new, effective therapeutic strategies for managing the non-motor symptoms of PD. As these symptoms significantly impact patients’ quality of life and are less responsive to conventional treatments, investigating the potential of propolis and its components offers an exciting opportunity for novel interventions in PD.

## Figures and Tables

**Table 1:** Relationship between non-motor symptoms of PD, related diseases investigated, effects of propolis, and active compounds identified.

Non-Motor Symptoms and Related Diseases Investigated	Common Pathophysiological Mechanisms of PD	Effects Observed with Propolis	Bioactive Compounds Identified	Ref.
Neuropsychiatric (Depression, anxiety, apathy): Major depression, generalized anxiety disorder, and chronic stress.	Early dopaminergic and serotonergic degeneration in the prodromal phase of PD; dysfunctions in the GABAergic and cholinergic systems; persistent neuroinflammation with an increase in pro-inflammatory cytokines (TNF-α, IL-1β, and IL-6); disruptions in the cortico-limbic-striato-thalamic circuits.	Significant reduction in depressive and anxiety symptoms in clinical and pre-clinical studies with propolis; reduction in neuroinflammation and pro-inflammatory cytokines; inhibition of the enzyme monoamine oxidase (MAO), increasing the availability of dopamine, serotonin, and noradrenaline; significant increase in BDNF brain expression; antioxidant protection, reducing neuronal oxidative stress; behavioral improvement in animal models of anxiety and depression induced by neuroinflammation and chronic stress.	Quercetin; Apienin; Chrysin; caffeic acid phenethyl ester (CAPE); Isochlorogenic acid A and B.	[10,31,32,33,34,35,36,37,38,39,40,41,42,43,44,45,46,47,48,49,50,51,52,53,54,55,56,57,58,59,60,61,62,63,64,65,66,67].
Cognitive deficits (Problems with memory, attention, and executive and visuospatial functions): age-related cognitive decline and mild cognitive impairment MCI), inflammatory and neurodegenerative processes associated with age and oxidative stress and neuropsychiatric disorders associated with cognitive decline.	Prevalence of cognitive impairment up to six times higher in individuals with PD than in the general population; increased risk of developing dementia; reduced levels of neurotransmitters (dopamine, serotonin, noradrenaline, and acetylcholine); persistent cerebral neuroinflammation (IL-1β, IL-6, and TNF-α); oxidative stress and neuronal degeneration, reducing brain expression of the neurotrophic factor BDNF, and neurogenesis.	Significant improvement in cognitive domains (memory, attention, and information processing) in elderly people treated with propolis; reduction in systemic inflammation (IL-1β and IL-6); increase in TGFβ1 associated with protection against cognitive decline; activation of the acetylcholine pathway; reduction in cerebral neuroinflammation; reduction in neuronal oxidative stress; inhibition of acetylcholinesterase; positive regulation of brain levels of neurotransmitters (dopamine, serotonin, and noradrenaline); significant increase in brain levels of BDNF, favoring neurogenesis and synaptic plasticity; reduction in brain protein aggregates induced by hyperhomocysteinemia; reduction in inflammatory markers, microglial activation, and neuroinflammation induced by sleep deprivation.	Quercetin; Apigenin; Chrysin; Caffeic acid phenethyl ester (CAPE).	[23,66,67,68,69,70,71,72,73,74,75,76,77,78,79,80,81,82].
Sleep Disorders (Insomnia, circadian rhythm disorders, sleep-disordered breathing [obstructive and central apnea], restless legs syndrome, behavioral REM sleep disorder, and excessive daytime sleepiness): sleep disorders related to alterations in the circadian cycle, obstructive sleep apnea, nocturnal intermittent hypoxemia, respiratory and inflammatory disorders associated with impaired sleep, and sleep deprivation induced by oxidative stress and neuroinflammation.	Reduced dopamine availability affecting sleep–wake dynamics and circadian rhythm; high prevalence of sleep disorders (60–90% of PD patients); significant presence of sleep apnea (obstructive and central) in PD patients, causing intermittent hypoxia, neuroinflammation, and neuronal damage; chronic inflammatory activation, increasing TNFα, IL-1β, and IL-6, which directly affect the internal biological clock; dysfunction in the neurotransmitter systems (dopaminergic, serotoninergic, cholinergic, and GABAergic), which are essential in sleep regulation; glial dysfunction compromising neuronal transmission and the synchronization of the circadian rhythm.	Improved sleep quality, reduced sleep latency and increased total sleep duration in animal models; significant reduction in cerebral oxidative stress and apoptosis induced by intermittent hypoxia and sleep deprivation; increased brain levels of dopamine and modulation of GABAergic activity, promoting sedation and improved sleep; reduction in microglial activation, neuroinflammation and pro-inflammatory cytokines; improvement in circadian parameters through antioxidant action and metabolic modulation, regulating the sleep–wake rhythm; positive interaction with GABA-A receptors, facilitating the induction and maintenance of non-REM sleep, also improving circadian patterns in animal models.	Apigenin; Quercetin; Cinnamic acid and derivatives (ferulic acid).	[83,84,85,86,87,88,89,90,91,92,93,94,95,96,97,98,99,100,101,102,103,104,105,106,107,108,109,110,111,112,113,114].
Gastrointestinal dysfunctions (Constipation, irritable bowel syndrome (IBS), changes in intestinal permeability, *Helicobacter pylori* infection, bacterial overgrowth (SIBO), and intestinal dysbiosis): functional constipation, IBS with a predominance of constipation, ulcerative colitis, chronic *Helicobacter pylori* infection, SIBO, dysbiosis, and chronic intestinal inflammation.	High prevalence of gastrointestinal dysfunction in PD (up to 80% of patients), often manifested years before motor symptoms; aggregation of α-synuclein in the enteric nervous system; chronic intestinal inflammation, increased intestinal permeability, dysbiosis with a reduction in short-chain fatty acid (SCFA)-producing bacteria; link between *Helicobacter pylori* infection and progression of motor symptoms in PD; high prevalence of bacterial overgrowth (SIBO) in PD patients; ascending theory from the gut to the brain (Braak’s theory).	Significant improvement in constipation and increased intestinal contractility via activation of cholinergic receptors (cholinomimetic effect); reduction in pain and frequency of abdominal pain in individuals with IBS associated with constipation; restoration of the integrity of the intestinal mucosa; reduction in intestinal inflammation via inhibition of the TLR4/NF-κB pathway; positive modulation of the intestinal microbiome and increased production of SCFAs, especially butyrate; improvement of intestinal barrier function by increasing tight junctions and positive regulation of proteins (ZO-1), via activation of AMPK and ERK1/2; inhibition of growth and production of urease by *H. pylori* and reduction in the bacterium’s virulence factors; reduction in the production of pro-inflammatory cytokines and intestinal oxidative stress; reduction in bacterial translocation and prevention of intestinal bacterial overgrowth (SIBO).	Pinocembrin; Quercetin; Kaempferol; Various phenolic and flavonoid compounds (Polyphenols); Caffeic acid phenethyl ester (CAPE).	[115,116,117,118,119,120,121,122,123,124,125,126,127,128,129,130,131,132,133,134,135,136,137,138,139,140,141,142,143,144,145,146,147,148,149,150,151,152,153,154,155,156,157,158,159,160].
Cardiovascular problems (Orthostatic and postprandial hypotension, cardiac dysautonomia, reduced heart rate variability (HRV), non-dipping, and cardiac dysfunction, including sudden unexpected death in PD [SUDPAR]): cardiac dysautonomia related to cardiovascular diseases (hypertension, heart failure, and coronary heart disease), dyslipidemia, hyperglycemia and type 2 diabetes mellitus, and metabolic alterations related to metabolic syndrome.	High prevalence of cardiovascular dysfunction in PD patients (approx. 80%); autonomic dysfunction with impairment of the sympathetic and parasympathetic nervous systems; reduced HRV and increased risk of orthostatic and postprandial hypotension; high prevalence of non-dipping; higher cardiovascular risk due to cardiac autonomic dysfunction; complex association between PD, cardiovascular diseases, and type 2 diabetes; chronic inflammation, mitochondrial dysfunction, and oxidative stress associated with cardiovascular complications; increased risk of sudden unexpected cardiac death in PD patients (SUDPAR).	Significant improvement in cardiac function and increase in heart rate variability (HRV); reduction in systemic blood pressure in hypertensive models; reduction in cardiovascular risk by modulating metabolic pathways and reducing oxidative stress; improved cardiac energy metabolism (increased glycolytic metabolism in the left ventricle); cardiovascular protection against autonomic alterations in animal models of PD; improvement of metabolic and inflammatory alterations associated with metabolic syndrome; reduction in arterial hypertension and improvement of the lipid and glycemic profile (e.g., effects of the AMPK and PPARs); decreased vascular inflammation and improved endothelial function, through AMPK activation and modulation of the ERK1/2 and p38 MAPK pathways; improved mitochondrial function and cardiac energy metabolism; reduction in cardiac apoptotic processes and prevention of cardiac arrhythmias.	Caffeic acid phenethyl ester (CAPE); Galangin; Quercetin.	[26,35,56,120,144,161,162,163,164,165,166,167,168,169,170,171,172,173,174,175,176,177,178,179,180,181,182,183,184,185,186,187,188,189,190,191,192,193,194,195,196,197,198,199,200,201,202,203,204,205,206,207,208,209,210].
Pain (Musculoskeletal pain, neuropathic, nociceptive and nociplastic pain, and pain related to stiffness and reduced mobility): rheumatoid arthritis, diabetic neuropathy, osteoarthritis, peripheral neuropathy, chronic inflammatory and neurogenic pain, and pain induced by oxidative stress and inflammation.	Dopaminergic dysfunction and altered pain pathways; increased systemic inflammation and oxidative stress; classification of pain in PD as nociceptive, neuropathic, and nociplastic; involvement of the opioid, serotonergic, and GABAergic systems; activation of inflammatory mediators, including COX-2 and NF-κB.	Significant reduction in neuropathic and inflammatory pain; inhibition of COX-2 and reduction in prostaglandin release; reduction in inflammation and oxidative stress via inhibition of the NF-κB pathway and activation of the antioxidant factor Nrf2; improvement of peripheral neuropathy and osteoarthritis by increasing the expression of enzymes.	CAPE; Chrysin; Quercetin; Apigenin	[211,212,213,214,215,216,217,218,219,220,221,222,223,224,225,226,227,228,229,230,231,232,233,234,235,236,237,238,239,240,241,242,243,244,245].

**Table 2:** Propolis bioactive compounds, mechanisms of action, potentially beneficial non-motor symptoms, and main associated references.

Bioactive Compound	Chemical Class	Main Mechanisms of Action	Non-Motor Symptoms Potentially Benefited
Quercetin	Flavonoids	Antioxidant, anti-inflammatory, increased BDNF, improved intestinal microbiota, and modulation of AKT1, IL6, MAPK1, and VEGFA	Neuropsychiatric, cognitive, sleep, gastrointestinal, cardiovascular, and pain
Apigenin	Flavonoids	GABAergic and dopaminergic modulation, anxiolytic, antioxidant, and neuroprotective	Neuropsychiatric, sleep, cognitive, and respiratory
Caffeic acid phenethyl ester (CAPE)	Phenolic	Potent anti-inflammatory, antioxidant, neuroprotective, cardioprotective, and modulator of energy metabolism via AMPK and PPAR	Cardiovascular, neuropsychiatric, pain, gastrointestinal, and cognitive
Chrysin	Flavonoids	Antidepressant, analgesic, anti-inflammatory, antioxidant, and serotonergic modulator	Neuropsychiatric, pain, and cognitive
Pinocembrin	Flavonoids	Anti-inflammatory, antioxidant, protection of the intestinal and pulmonary barrier, and immune modulation	Gastrointestinal and respiratory
Galangin	Flavonoids	Metabolic modulation, improvement of endothelial function, reduction in cardiovascular inflammation, and modulation of the angiotensin II/TGF-β pathway	Cardiovascular and gastrointestinal
Cinnamic acid and derivatives (e.g., ferulic acid)	Phenylpropenoic acid	Antioxidant, anti-inflammatory, circadian rhythm regulation, and GABAergic agonist activity	Sleep disorders
Kaempferol	Flavonoids	Modulation of AKT1, IL6, MAPK1, and VEGFA; antioxidant; anti- inflammatory; potential sleep regulator	Sleep and neuropsychiatric disorders

**Table 3:** Key mechanisms underlying the beneficial effects of propolis with references.

Key Mechanisms	Related Compounds	Ref.
Reduction in inflammation (TNF-α, IL-6, IL-1β, NF-κB, and TLR4)	CAPE, Quercetin, Pinocembrin, Chrysin, Galangin	[54,81,129,143,184,233]
Antioxidant activation via Nrf2/HO-1 and reduction in oxidative stress	CAPE, Quercetin, Chrysin, Galangin, Apigenin	[222,224,234,242,244,245,246,247]
Modulation of energy metabolism (AMPK and PPAR) and reduction in cardiovascular and metabolic risk	CAPE, Galangin, Quercetin	[56,189,190,191,192,193,194,195,196,197,198,199,200,201,202,203,204,205]
Inhibition of monoamine oxidase (MAO), increase in dopamine and serotonin, and neuroprotection	CAPE, Apigenin, Quercetin	[58,104,105,106,107]
Increased BDNF and neurogenesis and improved synaptic plasticity and neuronal protection	Apigenin, CAPE, Quercetin, Isochlorogenic acid A and B	[59,65,66,67]
Improved intestinal barrier function, modulation of the intestinal microbiome, and increase in SCFA	Pinocembrin, Quercetin, Polyphenols	[136,143,144,145,146,149]
Inhibition of monoamine oxidase (MAO) and increase in brain neurotransmitters (dopamine, serotonin, and noradrenaline)	CAPE, Apigenin, Quercetin, Chrysin	[58,59,63,64]

## Abbreviations

PD	Parkinson’s disease
BDNF	Brain-derived neurotrophic factor
REM	Rapid Eye Movement
SAS	Sleep-Apnea Syndrome
CREB	cAMP Response Element Binding
GABA	Gamma-Aminobutyric Acid
CA	Cinnamic Acid
IBS	Irritable Bowel Syndrome
SCFA	Short-Chain Fatty Acids
AMPK	Active Adenosine Monophosphate-Activated Protein Kinase
cGMP	Cyclic Guanosine Monophosphate
PKG	Protein Kinase G
KATP	Adenosine Triphosphate-Sensitive Potassium
ERK 1/2	Extracellular Signal-Regulated Kinases
IκBα	NF-kB Inhibitor Alpha
SIBO	Small Intestinal Bacterial Overgrowth
SUDPAR	Sudden Unexpected Death in Parkinson’s Disease
NO	Nitric Oxide
CAPE	Caffeic Acid Phenethyl Ester
SARS-Cov-2	Severe Acute Respiratory Coronavirus 2
COVID-19	Coronavirus 19
NF-kB	Nuclear Factor Kappa
COX-2	Cyclooxygenase 2
PGE-2	Prostaglandin E2
TGF-β	Transforming Growth Factor Beta
Nrf2	Nuclear Factor Erythroid 2-Related Factor 2
IL-6	Interleukin-6
IL-IB	Interleukin-1Beta
IL-8	Interleukin-8
TNF- α	Tumor Necrosis Factor Alpha
IFN- γ	Interferon-Gamma
IKB-kinase	Inhibitory Kappa B Kinase Beta
MAPK	Mitogen-Active Protein Kinase
JAK-STAT	Janus Kinase-Signal Transducers and Activators of Transcription
NRF2/Keap1-ARE	Nuclear Factor Erythroid 2-Related Factor/Kelch-Like ECH-Associated Protein 1-Antioxidant Response Element
HO-1	Heme Oxygenase-1
NQO1	NAD(P)H Quinone Dehydrogenase 1
GPx	Glutathione Peroxidase
GR	Glutathione Reductase
CAT	Catalase
PGC-1α	Peroxisome Proliferator-Activated Receptor Gamma Coactivator 1-Alpha
STAT3	Signal Transducer and Activator of Transcription 3
M1/M2	Macrophages
HMGB1	High-Mobility Group Box 1 Protein
DC	Dendritic Cell
CD4+T	T-Lymphocytes
Trag cell	Regulatory T Cells
FOXP3	Forkhead Box P3
TLR2/TRL4	Toll-Like Receptor 2/4
MyD88	Myeloid Differentiation Primary Response 88
NLRP3	Nucleotide-Binding Domain Leucine-Rich-Containing Family Pyrin Domain-Containing-3
Th cell	Helper T Cells
NK cell	Natural Killer Cell
B cells	B Lymphocyte Cells
AKt	Protein Kinase B
mTOR	Mammalian Target of Rapamycin
sre	Sterol Response Element
